# Study on the Impact of Microscopic Pore Structure Characteristics in Tight Sandstone on Microscopic Remaining Oil after Polymer Flooding

**DOI:** 10.3390/polym16192757

**Published:** 2024-09-29

**Authors:** Ling Zhao, Xianda Sun, Huili Zhang, Chengwu Xu, Xin Sui, Xudong Qin, Maokun Zeng

**Affiliations:** 1 College of Computer and Information Technology, Northeast Petroleum University, Daqing 163318, China; mirror_zl@nepu.edu.cn (L.Z.); zhl_0920@stu.nepu.edu.cn (H.Z.); zmk55678@163.com (M.Z.); 2 State Key Laboratory of Continental Shale Oil, Northeast Petroleum University, Daqing 163318, China; sunxianda@nepu.edu.cn (X.S.); 18814508221@163.com (X.S.); qinxudong@stu.nepu.edu.cn (X.Q.)

**Keywords:** microscopic pore structure, remaining oil type, pore size, coordination number, pore–throat ratio, wettability

## Abstract

As a non-renewable resource, oil faces increasing demand, and the remaining oil recovery rates in existing oil fields still require improvement. The primary objective of this study is to investigate the impact of pore structure parameters on the distribution and recovery of residual oil after polymer flooding by constructing a digital pore network model. Using this model, the study visualizes the post-flooding state of the model with 3DMAX-9.0 software and employs a range of simulation methods, including a detailed analysis of the pore size, coordination number, pore–throat ratio, and wettability, to quantitatively assess how these parameters affect the residual oil distribution and recovery. The research shows that the change in the distribution of pore sizes leads to a decrease in cluster-shaped residual oil and an increase in columnar residual oil. An increase in the coordination number increases the core permeability and reduces the residual oil; for example, when the coordination number increases from 4.3 to 6, the polymer flooding recovery rate increases from 24.57% to 30.44%. An increase in the pore–throat ratio reduces the permeability and causes more residual oil to remain in the throat; for example, when the pore–throat ratio increases from 3.2 to 6.3, the total recovery rate decreases from 74.34% to 63.72%. When the wettability changes from oil-wet to water-wet, the type of residual oil gradually changes from the difficult-to-drive-out columnar and film-shaped to the more easily recoverable cluster-shaped; for example, when the proportion of water-wet throats increases from 0.1:0.9 to 0.6:0.4, the water flooding recovery rate increases from 35.63% to 51.35%. Both qualitative and quantitative results suggest that the digital pore network model developed in this study effectively predicts the residual oil distribution under different pore structures and provides a crucial basis for optimizing residual oil recovery strategies.

## 1. Introduction

Petroleum, often referred to as the “blood of industry,” is an essential energy source for the economic and social development of human society, supporting sustainable economic growth. With the acceleration of economic development, the demand for petroleum continues to rise, highlighting the growing disparity between petroleum supply and national material needs. In China, despite primary and secondary oil recovery efforts, 60% to 70% of the original oil remains underground. Therefore, measures such as expanding the swept volume are needed to further enhance oil recovery rates. Due to the high heterogeneity of oil reservoirs, polymer flooding has proven to be an effective enhanced oil recovery technique [1,2]. Currently, the microscopic mechanisms of oil recovery are mainly investigated through laboratory core experiments. The theoretical foundation for improving recovery rates still relies on macroscopic theories based on Darcy’s law. Although Darcy’s law provides a description of fluid macroscopic flow, it does not accurately represent the pore size within rock pore spaces. Consequently, simulating the microscopic pore structure is crucial for understanding the flow mechanisms [3,4].

According to the extensive experimental research conducted by Chen Zhang, Deyu Zhu, and others [5], it is indicated that tight oil is mainly hosted in intragranular and intergranular pores, and the oil is distributed in fine pores and throats. At the same time, it is also pointed out that fractures play an important role in establishing the connectivity between pores and throats, which is conducive to the accumulation of tight oil. These research contents regarding pores provide an important basis for an in-depth understanding of the characteristics of tight oil reservoirs and the distribution of oil and gas in this area. Meanwhile, the sampling and investigation carried out by Chen Zhang and Dong-Dong Liu [6] in the Junggar Basin show that the genetic mechanism of natural fractures has a significant impact on the accumulation of shale oil, thus confirming the influence of the microscopic pore structure on residual oil from multiple angles and aspects.

Salter and Jerauld, through extensive experimental research, have demonstrated that the ratio of the pore radius to the average throat radius connected to it is a significant factor influencing wettability hysteresis. Additionally, the sizes of the pores and throats affect the shape of the relative permeability curves. Building on this foundation, numerous scholars, including Miller and Lowry, have investigated the topological characteristics of pore network models as well as the sizes of pores and throats, and explored their interrelationships [7]. In 1990, Mohammadi and colleagues proposed a pore network model consisting solely of pores and throats with numerous branches. This model is highly flexible and allows for structural adjustments by varying the coordination number [8]. In 1992, Bryant et al. applied a process simulation method to construct a network model that closely approximates the pore space of real rock cores and successfully predicted parameters such as absolute permeability and capillary pressure [9]. Subsequently, Bakke and Ren improved upon Bryant’s method by generating a network model using process simulation to predict the flow mechanisms in sandstones [10]. In 1995, Friedman et al. employed a cubic pore network model to calculate the model’s diffusion coefficient [11]. Dixit and colleagues, in 1999, used a regular topological pore network model to investigate the effects of wettability on enhanced oil recovery during waterflooding [12]. In 2008, Erzeybek and Akin developed a regular pore network model for carbonate rocks and simulated two-phase flow processes. Their results indicated that the relative permeability curves for fractures were similar in shape to those for oil–water systems [13]. In 2009, Hassanizadeh and Raoof introduced a new method for constructing pore network models, which more accurately represents the structural characteristics of real core pore spaces [14]. Although regular topological pore network models, which differ significantly from the pore spaces of actual cores, do not fully reflect the spatial features of rock cores, the diverse representation of pore and throat elements and their size assignments offer varied modeling approaches.

In many oil fields in China, the extraction methods have gradually transitioned from water flooding to polymer flooding and composite flooding, which has also led to changes in the distribution patterns of residual oil from those observed after water flooding to those after composite flooding [15]. During water flooding, the high mobility and low viscosity of water result in a poor oil displacement efficiency and leave a significant amount of residual oil underground [16]. In contrast, polymer flooding primarily aims to increase the swept volume during the oil displacement process, thereby displacing residual oil trapped in the pore spaces. However, this method may not displace oil completely, and, in areas with relatively low local permeability, the volume of residual oil remaining underground can still be considerable [17].

To assess polymer flooding efficiency, dynamic data from oil–water wells and logging interpretation curves are typically analyzed. By comparing core monitoring data before and after polymer flooding and examining the residual oil distribution, we can understand the changes in reservoir properties and residual oil patterns. The logging interpretation curves clearly show the extent of the polymer flood sweep within the injection–production range, with very low and highly dispersed residual oil saturation, indicating that polymer flooding is superior to water flooding. Outside this range, water flooding conditions persist with significant residual oil patches. The potential can be tapped using original well networks for additional holes. Despite the better displacement efficiency in central microfacies zones, residual oil remains substantial due to the initially higher reservoir thickness and oil saturation. Peripheral zones show a poorer oil recovery and higher residual oil volumes. Vertical analysis reveals higher water saturation in the upper and middle water-flooded layers, with residual oil saturation reduced in target zones after polymer flooding, which enhances recovery efficiency, especially in thin layers under one meter [18].

Among the methods for enhancing oil recovery in tight oil reservoirs, water flooding, as a relatively low-cost and mature technique, is widely used but has limited effectiveness in low-permeability tight oil reservoirs, prone to water channeling. In contrast, carbon dioxide flooding has the advantage of dissolving crude oil and reducing viscosity, but it requires high temperatures and pressures in the reservoir and has a high initial investment cost. Polymer flooding can improve the oil displacement efficiency and delay water breakthrough, but polymers are prone to degradation under high-temperature and high-salt conditions, and the injection is difficult. Surfactant flooding can reduce the oil–water interfacial tension and help mobilize bound oil, but the high cost and limitations of geological conditions affect its application effect. Gas alternating injection can prevent gas channeling and improve recovery by alternately injecting gas and water, but the operation is complex and the cost is high. Steam flooding can effectively heat the oil reservoir and improve the fluidity of crude oil, which is suitable for tight oil reservoirs, but its high energy consumption and environmental issues are also challenges that need to be considered [19].

In summary, the current research on the content and types of residual oil after hydraulic fracturing predominantly relies on physical models. While physical models, which directly simulate real-world conditions and allow for intuitive observation and manipulation through visual and tactile means, facilitate the understanding of complex system behaviors and principles, they often require actual materials and equipment for construction. This can involve significant economic costs and resource consumption. Additionally, the construction and testing of physical models can be time-consuming, particularly when experimental conditions or boundary conditions need to be changed, requiring the re-construction or adjustment of the model, thus extending the experimental cycle. Moreover, physical models may be influenced by experimental procedures and environmental factors, which can affect the precision and repeatability of the results compared to digital models. Therefore, there is a need to explore digital models as an alternative to traditional physical models. Digital models offer the advantage of quickly adjusting parameters and conditions, facilitating the study of fluid flow behaviors under different conditions. Based on precise mathematical formulae and algorithms, digital models can provide reproducible and consistent results, simulating fluid movement and distribution at a microscopic scale, thereby offering valuable insights for reservoir evaluation. According to the literature, an increasing number of researchers have proposed digital models, but these models typically focus on specific aspects, such as coordination number, porosity, and wettability, for single-variable predictions.

This paper constructs a digital pore network model and uses 3DMAX software to visualize the state of the model after the flooding process. It examines the impact of pore structure parameters, such as the pore size, coordination number, pore–throat ratio, and wettability, on the efficiency of water and polymer flooding. It quantitatively studies the changes in the proportion of oil-saturated pores before and after flooding, visualizes and classifies residual oil types in the model images, and analyzes how variations in pore structure parameters influence the distribution and types of residual oil. The development and simulation of the pore network model have been crucial for understanding the impact of pore structure parameters on oil recovery, providing significant theoretical and technical support for oil field development.

## 2. Network Construction and Performance Parameters

### 2.1. Construction of the Digital Pore Network Model

Rocks formed under various conditions over long periods in the Earth’s crust contain pores. The size and shape of these pores vary depending on the geological age of the rocks [20]. Pores refer to the relatively large storage spaces between solid particles in the rock framework that are not occupied by solid materials. Narrow connecting spaces between pores are known as throats. Pore structure parameters are those that characterize the features of these pores. The oil and gas studied in this paper are stored and flow through these pores and throats. In constructing the digital core model, this study primarily considers four pore structure parameters: hte pore and throat size distribution, coordination number, throat shape, and pore–throat ratio [21,22,23]. The foundational data for constructing the pore network model in this study primarily originate from mercury intrusion experiments. The pore and throat size distributions are obtained through conventional mercury intrusion and constant-rate mercury intrusion techniques. Constant-rate mercury intrusion involves injecting mercury into reservoir rocks at a very low and constant rate, with the process approximated as quasi-static. Due to the varying pore sizes within the rock, the capillary forces experienced by the mercury during flow differ accordingly. As mercury moves from larger pores into smaller throats, the capillary pressure increases gradually. Upon breakthrough, there is a sudden drop in pressure, which allows for the measurement of throat size distribution based on this pressure peak. Table 1 provides the pore structure parameter data for five core samples from an oil reservoir in the Daqing Oilfield.

#### 2.1.1. Pore Distribution Characteristics

The commonly used distribution functions for analyzing pore and throat sizes include the normal distribution [24] and the Weibull distribution [25]. When simulating the flow behavior within a reservoir, the shape of the pore and throat size distribution curves in the model can be determined based on the mercury intrusion curve data from actual rock samples of the reservoir [26].

**Figure 1:**
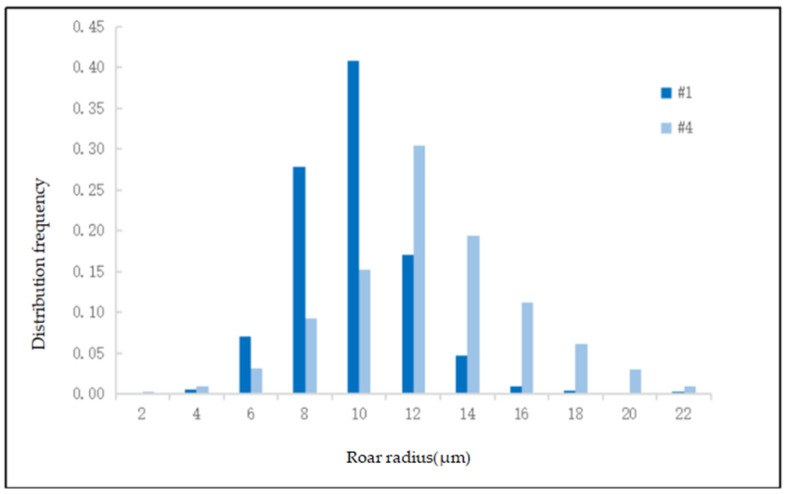
Distribution of throat radius.

For reservoir rocks, the macroscopic factors affecting porosity and permeability are primarily porosity and permeability itself, while the microscopic factors include the size and distribution frequency of pores. These parameters are fundamental for constructing pore models. Due to variations in size, shape, and development, acquiring related data is challenging. This study utilizes capillary pressure peak values obtained from mercury intrusion experiments to provide the distribution frequency of throat radii. Table 2 and Figure 1 present the throat distribution frequencies for core samples 1# and 4#.

#### 2.1.2. Geometric Characteristics of Pore–Throat Spaces

Parameters used to describe the pore network space include the average pore radius [27], average throat radius [28], coordination number [29], pore-to-throat ratio [30], shape factor [31], pore–throat radius distribution frequency [32], and other related parameters.

(1) Coordination Number: This refers to the number of throats connected to each pore. Using CT scans of core samples and image processing, the coordination number is determined.

(2) Pore-to-Throat Ratio: In porous media, the wider sections of the pore space are designated as pores, while the narrower sections are referred to as throats. The ratio of the pore radius to the throat radius is defined as the pore-to-throat ratio. In the constructed model, throat radii are assigned using a stochastic method. There exists a specific relationship between the pore radius and the throat radius, as described by the following formula [33]:

(1) $$r_{p}=\sum_{i=1}^{n}r_{ti}/n\times \alpha$$

In the formula, $r_{P}$ represents the pore radius, $r_{t}$ denotes the throat radius, and α signifies the average pore-to-throat ratio.

This formula provides the basis for assigning values to pores and throats in the constructed model, ensuring that the assigned pore radius cannot be smaller than the radius of the connected throat.

(3) Shape Factor: This refers to the dimensionless ratio of the volume of a geometric body to its surface area raised to a power. The true shapes of pore spaces are highly variable and complex, making their precise representation in limited computational and storage capacities challenging. Consequently, simplified geometric shapes and their combinations are used for approximation. This study employs regular geometric shapes such as circles, squares, and triangles to simulate different cross-sectional shapes of pore and throat spaces in the pore network model. In pore and throat spaces containing both square and triangular components, the simultaneous presence of water and oil phases is permitted to model fluid flow conditions in corner regions. The shape factor (*G*) is introduced to quantify this [34];

(2) $$G=\frac{VL}{{A_{s}}^{2}}$$

In the formula, As represents the surface area of the pore and throat volume element; *V* denotes the volume of the volume element; and *L* signifies the length of the volume element.

**Figure 2:**
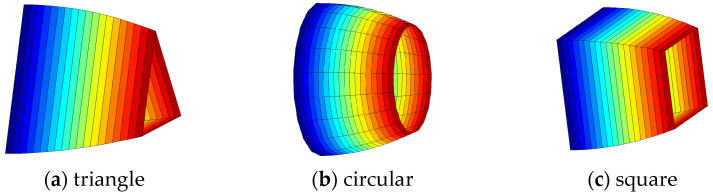
Schematic diagram of asymmetric throat change.

Currently, the throats in the constructed pore network models are predominantly represented as equidimensional cylindrical bodies. In the model presented in this paper, the throat radius exhibits an asymmetric sinusoidal variation, as shown in Figure 2.

**Figure 3:**
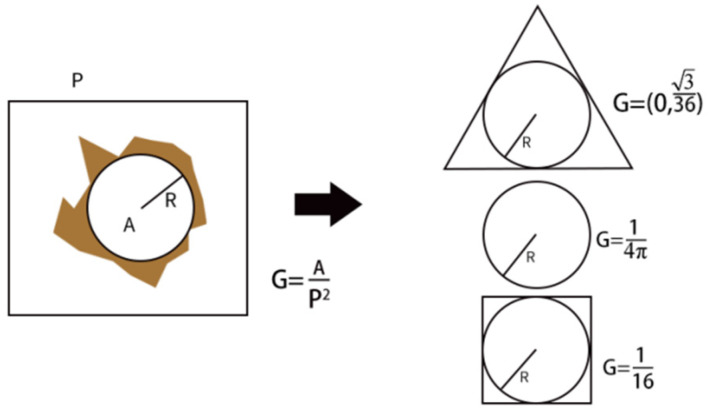
Shape factor of pore throat spatial section in pore network model.

Using the regular geometric shapes depicted in Figure 3, Equation (2) is simplified to Equation (3):

(3) $$G=\frac{A}{P^{2}}$$

In the equation, A represents the cross-sectional area of the pore and throat, while P denotes the perimeter of the cross-section of the pore and throat.

When the cross-sectional shape is an equilateral triangle, (*G* = 0.0481); when the cross-sectional shape is a square, (*G* = 0.0625); and when the cross-sectional shape is a circle, (*G* = 0.0796).

All throats connected to a given pore converge at the center of the pore, and the inscribed radius between the pore and its connected throats is calculated as follows:

(4) $$r=\left(\frac{R_{p}+R_{t}}{2}\right)-\left(\frac{R_{p}-R_{t}}{2}\right)\cos\left(\frac{2\pi x}{l_{p}+l_{t}}\right)$$

In the equation: $R_{P}$ represents the pore radius; $R_{t}$ denotes the throat radius; x indicates a specific position along the throat; and $l_{t}$ is the throat length [35].

#### 2.1.3. Construction and Visualization of the Pore–Throat Network Model

**Figure 4:**
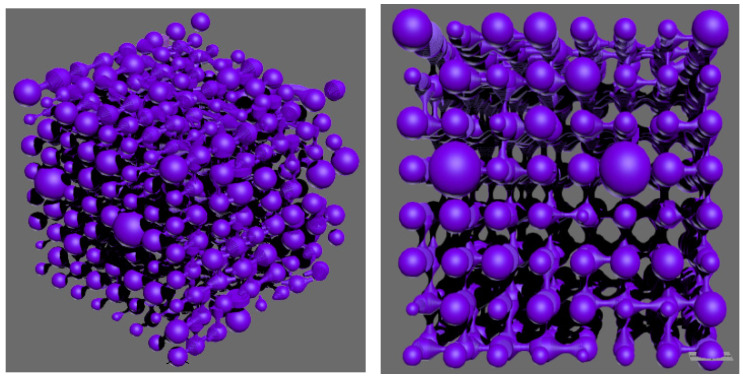
Digital pore and throat network model.

When constructing a digital model, selecting appropriate node parameters is crucial. Too few nodes can compromise the accuracy of the simulation, while too many nodes can lead to a polynomial increase in both the time and space complexity of the model [36]. After extensive trial and error and considering experimental conditions, a final choice of 8 × 8 × 8 = 512 nodes was made. To account for the non-uniform diameters of pore–throat spaces, a digital three-dimensional network model was developed featuring asymmetric corrugated tubular pore–throats that accurately reflect the actual pore structure of the rock core. A visualization of the constructed digital pore model was achieved using 3Dmax software. Parameters such as node count, and physical coordinates of each pore and throat, as well as pore–throat sizes and lengths were converted into a script format compatible with 3Dmax for input and execution, as shown in Figure 4. In the three-dimensional space, asymmetric corrugated tubular pore–throats interconnect in a complex manner across the three coordinate axes, presenting challenges for subsequent residual oil analysis. To address the non-uniformity of the throats, the pore–throats in a two-dimensional plane were randomly rotated between 0 and 120 degrees. By adjusting these angles to maximize connectivity between pore–throats in the two-dimensional plane, further analysis of the residual oil morphology was facilitated, enabling a more detailed study of residual oil patterns.

### 2.2. Performance Parameters

#### 2.2.1. Calculation of Oil Saturation

In the simulation of oil saturation within the digital pore model, to ensure the continuous observation of the simulation process, the oil saturation is recalculated and refreshed after each step of the saturation process [37]. Upon completion of the oil saturation simulation, the oil saturation is determined as the ratio of the total volume of oil within the model to the total volume of the pore model. The calculation of oil saturation is represented by Formula (5):

(5) $$S_{o}=\frac{\sum_{i=1}^{n_{1}}V_{t}+\sum_{j=1}^{n_{2}}V_{p}}{V}$$

In the formula: 

$V_{p}$—the volume of pores invaded by oil, μm^3^;

V—the pore volume of the model, μm^3^;

$n_{1}$, $n_{2}$—the volumes of the invaded throats and the total pore volume within the model [38].

For the throat volume Vt, when it contains only the oil-phase fluid, $V_{t}=Cr_{t}^{2}L$; when the oil-phase fluid is located solely at the central position of the throat, $V_{t}=A_{cen}L$. Formula (5) can be transformed into Formulae (6) and (7):

(6) $$S_{o}=\frac{\sum_{i=1}^{n_{1}}Cr_{t}^{2}L+\sum_{j=1}^{n_{2}}V_{p}}{V}$$

(7) $$S_{o}=\frac{\sum_{i=1}^{n_{1}}A_{cen}L+\sum_{j=1}^{n_{2}}V_{p}}{V}$$

During the simulation of dynamic oil saturation, if the oil saturation value does not reach the target value, the simulation process is iteratively executed until the oil saturation value in the model stabilizes. If the oil saturation value still does not meet the target after stabilization, adjustments must be made to the initial parameters of the simulation program, such as increasing the initial pressure or modifying other parameters.

In physical simulation waterflood experiments using real core samples, the criterion for the end of waterflooding is when the water cut reaches 98%. In the digital pore network model, the cumulative oil production and cumulative water production over a series of consecutive simulation time steps are calculated to determine the oil saturation and water cut of the model. The waterflooding process is considered complete when two consecutive measurements of the 98% water cut are observed.

#### 2.2.2. Performance Parameters of Polymer Solution Systems

The viscoelasticity of a polymer solution refers to the distinct viscous and elastic behaviors exhibited by the polymer [39]. Depending on the force conditions, polymer solutions display varying viscous and elastic properties [40]. During seepage in porous media, polymer molecules are subjected to stress, resulting in two different types of flow: viscous flow, described by the non-Newtonian viscosity of the polymer solution, and viscoelastic flow, characterized by the viscoelastic properties of the polymer solution [41]. When polymer molecules experience shear, from the perspective of vector analysis and computation, a normal stress is generated in a direction perpendicular to the shear force. In the first Newtonian region, the viscosity value is approximately constant, known as the zero-shear viscosity. During the steady-state shear and small-amplitude oscillatory shear flow stages, shear thinning occurs in certain polymer solutions, where the viscosity decreases gradually as the shear rate increases.

The rheological behavior of non-Newtonian fluids is often characterized by the Cross equation [42]:

(8) $$\frac{\eta -\eta_{\infty }}{\eta_{0}-\eta_{\infty }}=(K\gamma {)}^{m}$$

In the equation:

$\eta_{0}$—viscosity asymptote at extremely low shear rates;

$\eta_{\infty }$—viscosity asymptote at extremely high shear rates;

K—time-dimensional constant;

m—dimensionless constant.

When $\eta <<\eta_{0}$ and $\eta >>\eta_{\infty }$, the Cross formula can be simplified to the following:

(9) $$\eta =\frac{\eta_{0}}{(K\gamma {)}^{m}}$$

After redefining the parameters, Equation (9) can be rewritten as the power-law equation shown in Equation (10):

(10) $$\eta =K\gamma^{n-1}$$

In the equation:

n—power-law exponent, dimensionless;

K—consistency coefficient, dimensionless;

η—viscosity, mPa⋅s;

γ—shear rate, s^−1^.

**Figure 5:**
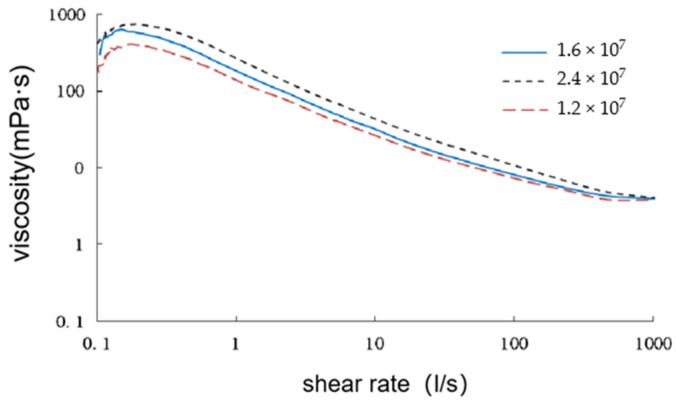
Curve of viscosity and shear rate under different molecular weight.

When simulating the seepage process and behavior of polymer solutions in porous media using a digital pore network model, the power-law function model represented by Equation (9) is employed to describe the viscosity of polymer solutions in the shear thinning region. The curve representing the relationship between the viscosity and shear rate can be obtained from experimental measurements, and, by regressing the data, the parameters K and n can be derived. These parameters are then substituted into Equation (10) to obtain the power-law index and consistency coefficient, allowing the calculation of the viscosity as a function of the shear rate for polymer solutions with different molecular weights and concentrations [43]. The variation curves of the viscosity and shear rate under different molecular weights are shown in Figure 5.

The viscoelasticity of polymers affects fluid flow patterns and flow resistance, making it crucial for the behavior in porous media. Relative to factors such as the pore throat size, porosity, and pore throat distribution, the viscoelasticity of polymers can significantly influence fluid dynamics. However, the specific degree of impact depends on the application scenario, and the influence of viscoelasticity is generally smaller compared to the microstructural characteristics such as the pore throat size and coordination number.

### 2.3. Experimental Data Validation

For cores #1, #3, and #4, saturate the digital pore network model with oil first, and then conduct polymer flooding experiments. Simultaneously, perform physical simulation experiments on cores #1, #3, and #4 to validate the simulated digital pore network model through polymer flooding experiments. Analyze the impact of pore structure parameters on residual oil distribution and recovery efficiency, and verify the accuracy and reliability of the simulation results.

1. Experimental Design: Select core samples with different pore structures to conduct polymer flooding experiments, measuring the oil recovery performance and residual oil distribution.

(1) Experimental Materials

Three core samples with different pore structure characteristics were selected, labeled as #1, #2, and #3. The porosities are 25.1%, 24.6%, and 26.3%, respectively, with permeabilities ranging from 39 to 623 mD, and a diverse distribution of pore throat sizes.

Use partially hydrolyzed polyacrylamide (HPAM) with a concentration of 1000 ppm, viscosity ranging from 30 to 35 mPa·s, and salinity controlled at 5000 mg/L.

It is then mineralized to match the reservoir formation water, with a salinity of 8000 mg/L to ensure consistency with reservoir conditions.

(2) Experimental Equipment

① Core holder;

② High-pressure injection pump;

③ Differential pressure sensor;

④ Constant-temperature device (set to 60 °C to simulate subsurface reservoir conditions);

⑤ Fluid collector;

⑥ Measurement equipment (including recovery rate tester and permeability measurement device).

(3) Experimental Procedure

① Clean and dry the selected three core samples, and measure their porosity, permeability, and pore–throat size distribution;

② Saturate the core samples with simulated water to ensure they reach water saturation, and determine the initial water saturation;

③ Place the core samples in the core holder and inject the simulated water at a constant rate of 0.5 mL/min until the water cut reaches 98%;

④ Record the recovery rate and pressure differential during the water injection phase, and analyze the residual oil distribution;

⑤ After completing the waterflood, start injecting the polymer solution at the same rate as during the waterflood until the outlet water cut reaches 98% again;

⑥ Record the recovery rate changes, pressure differential, and residual oil saturation during the polymer flooding process, and observe the residual oil distribution.

2. Data Analysis: Compare the experimental results with the simulation results to assess the credibility of the simulation outcomes. The specific experimental results are shown in Table 3.

3. Data Analysis: List the range of errors between the experimental and simulation results, analyze the sources of these errors, and improve the model accordingly.

(1) Waterflood Recovery Rate Analysis

Core #1: The numerical model predicts a waterflood recovery rate of 45.03%, while the physical model measures 47.07%, resulting in a 2.04% difference;

Core #3: The numerical model predicts a waterflood recovery rate of 46.56%, whereas the physical model measures 49.12%, leading to a 2.56% difference;

Core #4: The numerical model predicts a waterflood recovery rate of 44.17%, compared to the physical model’s 46.22%, with a 2.05% difference.

Overall, the numerical model predicts slightly lower waterflood recovery rates compared to the physical model, with an error margin of around 2%. The waterflood recovery rates from both the numerical and physical models are relatively consistent.

(2) Polymer Flood Recovery Rate Analysis

Core #1: The numerical model predicts a polymer flood recovery rate of 23.69%, while the physical model measures 24.65%, resulting in a 0.96% difference;

Core #3: The numerical model predicts a polymer flood recovery rate of 21.49%, compared to the physical model’s 22.76%, leading to a 1.27% difference;

Core #4: The numerical model predicts a polymer flood recovery rate of 23.68%, versus the physical model’s 25.58%, with a 1.90% difference.

For polymer flood recovery rates, the error between numerical and physical models is relatively small, ranging from 1% to 2%, indicating a high level of agreement between the numerical predictions and experimental results.

(3) Total Recovery Rate Analysis

Core #1: The numerical model predicts a total recovery rate of 68.72%, whereas the physical model measures 71.71%, resulting in a 2.99% difference;

Core #3: The numerical model predicts a total recovery rate of 68.05%, compared to the physical model’s 71.88%, leading to a 3.83% difference;

Core #4: The numerical model predicts a total recovery rate of 67.85%, versus the physical model’s 71.76%, with a 3.91% difference.

For total recovery rates, the error between the numerical predictions and physical model results is approximately 3%, which is slightly lower, but the overall trend is consistent.

4. Experimental Summary

The average absolute relative deviation (AARD%) is an indicator used to measure the difference between experimental results and simulation results, and is often used to evaluate the accuracy of numerical models [44]. The calculation formula of AARD% is as follows:

(11) $$AARD\%=\frac{1}{N}\sum_{i=1}^{N}|\frac{ExperimentalValue_{i}-SimulatedValue_{i}}{ExperimentalValue_{i}}|\times 100$$

In the equation: 

*N* is the number of data points;

ExperimentalValue is the measured value from experiments;

SimulatedValue is the predicted value from the numerical model.

A smaller AARD% indicates that the difference between the numerical simulation results and the experimental results is smaller, and the prediction accuracy of the model is higher. When the AARD% is greater than 10%, it is generally considered that the error is relatively large, and the model may need to be further improved. When the AARD% is less than 10%, it is often considered that the error is within a reasonable range, indicating that the prediction ability of the model is better.

According to the above data analysis, the AARD% of the water flooding recovery rate is 4.66%, the AARD% of the polymer flooding recovery rate is 5.63%, and the AARD% of the total recovery rate is 4.98%. According to the common comparison standards for petroleum engineering simulations and experiments, an average absolute relative deviation (AARD%) usually less than 10% is considered to be within a reasonable error range, especially in the simulation of complex reservoirs. Therefore, the calculated AARD% of the water flooding recovery rate (4.66%), the polymer flooding recovery rate (5.63%), and the total recovery rate (4.98%) are all below 10%, which is within the acceptable error range. This means that the numerical simulation results are highly consistent with the experimental results, and it can be considered that the simulation model has a good prediction ability.

## 3. The Impact of Pore Size Distribution on Various Types of Remaining Oil

### 3.1. The Impact of Pore Size Distribution on Oil Displacement Efficiency

**Figure 6:**
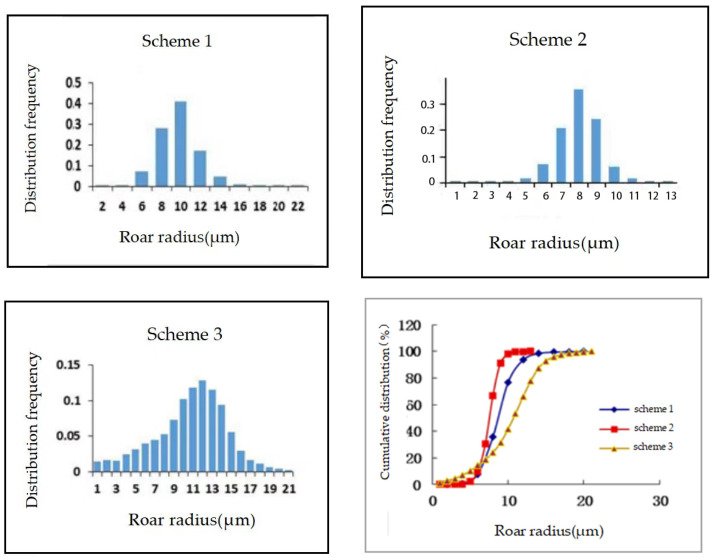
Throat distribution frequency curve and cumulative distribution curve of different schemes.

The throat radius is a fundamental parameter in digital pore models, and the recovery rate is closely related to the size of the throat radius. In this study, the models were established using the throat radius distribution frequencies of three actual core samples. Under the condition that other parameters remain constant, the effect of pore size distribution on recovery efficiency was investigated. Figure 6 shows the frequency distribution and cumulative frequency distribution of throat radius for the three schemes.

As shown in Table 4, the table lists the water flooding recovery rate, polymer flooding recovery rate, and total recovery rate for the three schemes. The results indicate that different pore sizes significantly impact the oil recovery efficiency during polymer flooding. Specifically, larger pore sizes can enhance the mobility of the polymer and the efficiency of oil recovery, thereby increasing the overall recovery rate.

**Figure 7:**
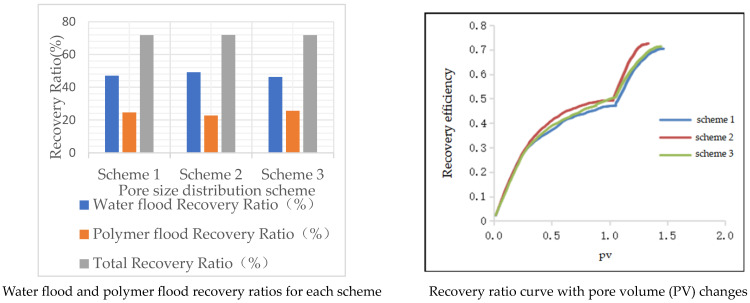
Model of different throat distribution frequency.

Figure 7 illustrates the recovery rates and the curves of recovery rates as a function of pore volume (pv) for different schemes. The permeability of Schemes 1 and 2 is similar but lower than that of Scheme 3. The pore throat distribution in Schemes 1 and 2 is relatively concentrated, with pores in the 6 μm~14 μm radius range accounting for 97%. In contrast, the pore throat distribution in Scheme 3 is more dispersed. The uneven pore throat distribution can lead to uneven fluid flow in the reservoir, increasing fluid retention in the rock. Regions with larger pore throats may allow fluid to flow quickly, while smaller pore throats may cause fluid blockage or difficulty in movement, making it challenging to efficiently extract oil from some reservoir areas, thereby reducing the overall recovery efficiency. The proportion of pore throats with a radius greater than 14 μm is close to 13%, which allows fluid to pass more easily through the rock, reducing flow resistance and thus increasing permeability. Therefore, the permeability of Scheme 3 is higher. Additionally, the water flooding recovery rate of Scheme 3 is lower because, compared to Schemes 1 and 2, Scheme 3 has more than 10% of pore throats with a radius less than 6 μm, and the oil in small pores is difficult to recover during water flooding, leading to a lower water flooding recovery rate for Scheme 3. Schemes 1 and 2 have higher water flooding recovery rates. The viscoelasticity of polymers can affect fluid movement in small pores, which significantly impacts Scheme 3, allowing it to recover more of the residual oil left after water flooding. Therefore, when comparing the polymer flooding recovery rates of Schemes 1, 2, and 3, Scheme 3 has the highest recovery rate.

### 3.2. Study on Residual Oil Distribution and Its Regularity When Pore Size Distribution Is Different

**Figure 8:**
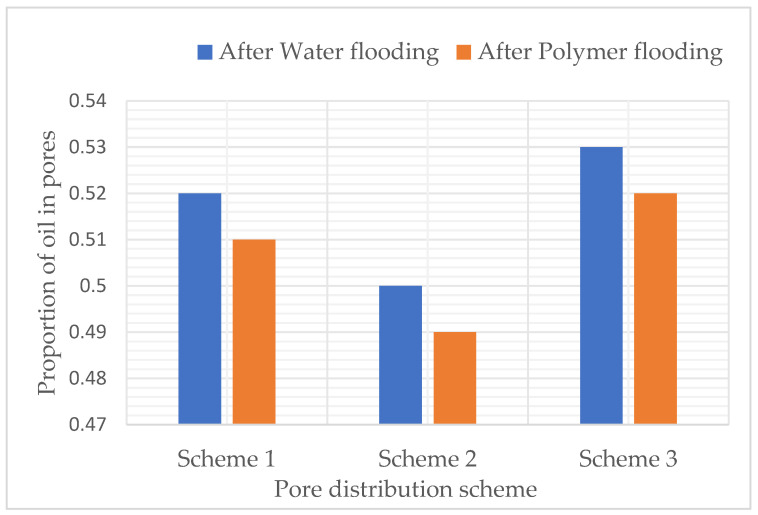
Oil-bearing ratio of pore channels after water flooding and polymer flooding under the pore size distribution scheme.

**Figure 9:**
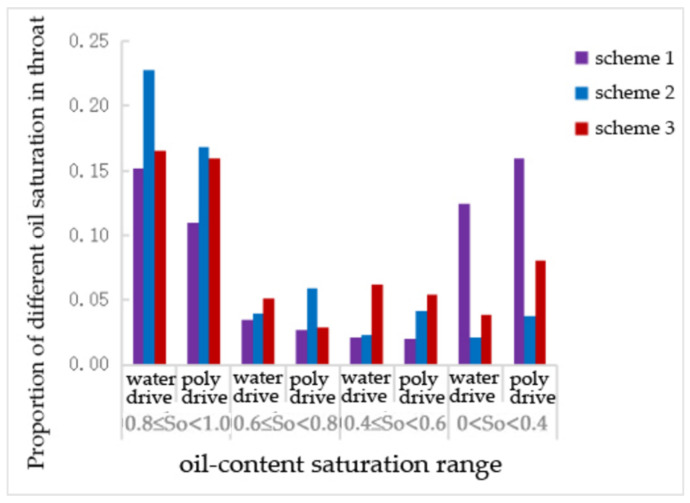
Proportion of throats with different oil saturations under the pore size distribution scheme.

**Figure 10:**
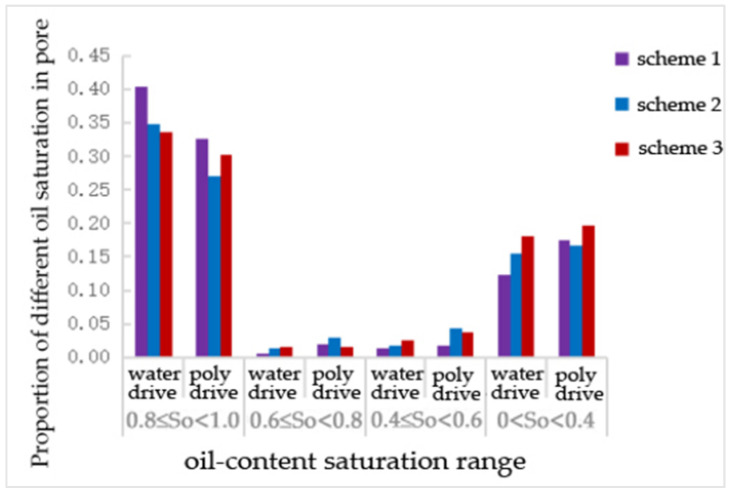
The proportion of pores with different oil saturations under the pore—size distribution scheme.

After water flooding and polymer flooding, the number of pores containing oil was statistically analyzed. Table 5, Table 6 and Table 7 and Figure 8, Figure 9 and Figure 10 show the proportion of different oil saturation levels in the pores after water flooding and polymer flooding. It can be seen that Scheme 3 has the highest number of pores with zero oil saturation after water flooding and the highest number of pores with zero oil saturation after polymer flooding. According to Table 5, the oil-bearing ratios of the pores after water flooding in Schemes 1, 2, and 3 are 0.38, 0.39, and 0.36, respectively, and the oil-bearing ratios after polymer flooding are 0.37, 0.38, and 0.35, respectively. It is evident that Scheme 3 has the lowest oil-bearing ratio and the highest recovery rate.

It can be observed that, compared to water flooding, the number of throats with an oil saturation of 10.8 decreased after polymer flooding, while the number of throats with other oil saturation levels increased to varying degrees. Specifically, the throats with an oil saturation of 0.8~0.6 decreased in Schemes 1 and 2, with reductions ranging from 22.2% to 44.3%, whereas the number of throats in this range increased by 46.8% in Scheme 3. The throats with an oil saturation of 0.60.4 decreased by 6.1% and 12.6% in Schemes 1 and 2, respectively, while increasing by 85.7% in Scheme 3. The number of throats with an oil saturation of 0.40 increased in all three schemes, with increases ranging from 28.4% to 108.3%. The number of throats with zero oil saturation changed by less than 3% across all schemes, indicating little to no change.

As shown in Table 6, compared to water flooding, the number of pores with an oil saturation of 1~0.8 decreased after polymer flooding in Schemes 1 and 3, while the number of other pores increased. Scheme 2 exhibited the opposite trend.

Table 7 shows that, compared to water flooding, the number of throats with an oil saturation of 0.8~1 decreased after polymer flooding in all schemes. Specifically, Scheme 3 saw a decrease of 6.25%, which is not as significant as the nearly 24% reduction in Schemes 1 and 2. The number of throats with an oil saturation of 0.6~0.8 decreased by 11% and 40% in Schemes 1 and 3, respectively, while increasing by 53% in Scheme 2. The number of throats with an oil saturation of 0.4~0.6 did not change significantly in Schemes 1 and 3 but increased by 100% in Scheme 2. The number of throats with an oil saturation of 0~0.4 increased to varying degrees across all schemes.

### 3.3. Study on Types and Quantitative Analysis of Residual Oil When Pore Size Distribution Is Different

**Figure 11:**
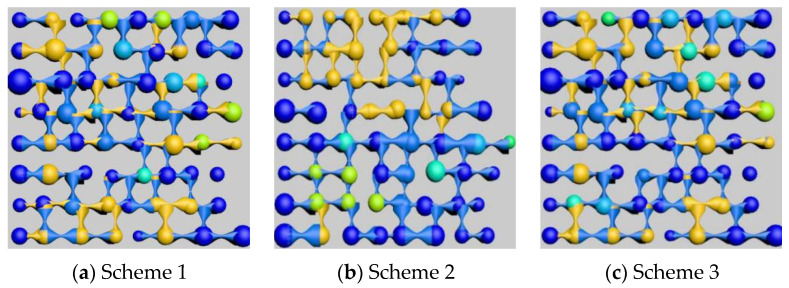
Influence of different throat radius distribution on remaining oil distribution.

**Figure 12:**
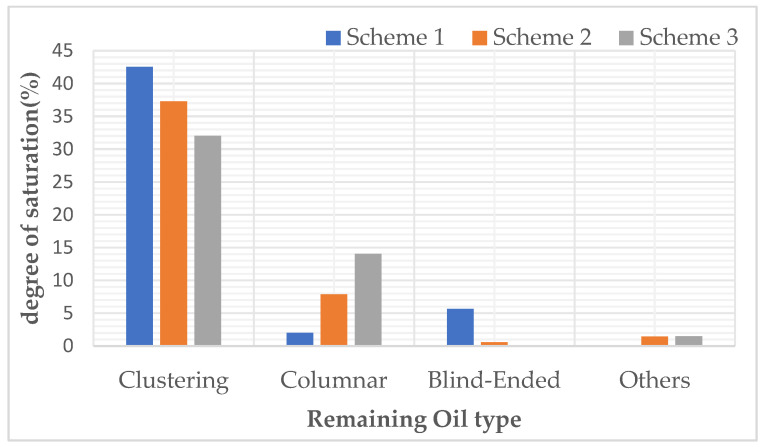
Influence of different throat radius distribution on remaining oil type.

After polymer flooding, oil distribution images of a specific cross-section were captured and rendered from models with three different throat radius distributions, as shown in Figure 11. Image (a) shows the residual oil distribution after polymer flooding in the model generated according to Scheme 1, image (b) corresponds to Scheme 2, and image (c) corresponds to Scheme 3. Table 8 and Figure 12 presents the results of the residual oil type identification and saturation statistics for each image.

The outcomes of the simulation indicate that alterations in the distribution of pore sizes lead to a diminution in the prevalence of clustered remaining oil, concurrently accompanied by an augmentation in the quantity of columnar remaining oil, with other remaining oil types manifesting stochastic fluctuations. This observed trend is attributed to the augmented permeability resultant from variations in the throat radius, which effectively promotes the displacement of sizeable aggregates of the clustered remaining oil into more dispersed morphologies.

## 4. The Impact of Coordination Number on Various Types of Remaining Oil

### 4.1. The Impact of Coordination Number on Various Types of Remaining Oil

**Figure 13:**
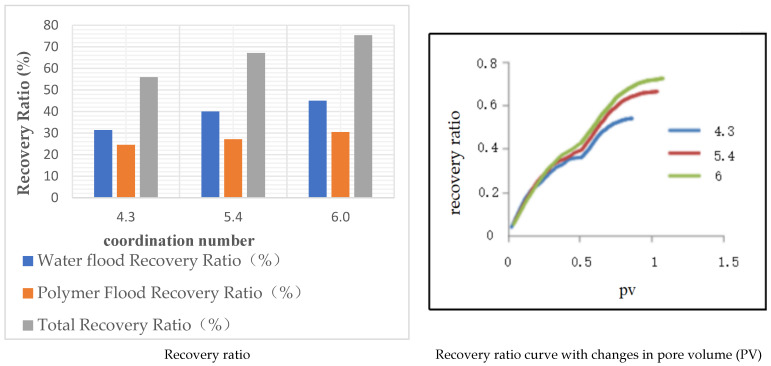
Models of different coordination numbers.

This study selected average coordination numbers of 4.3, 5.4, and 6 based on Scheme 2 to construct digital pore models under the same conditions for other parameters. Water flooding was concluded when the pore volume (pv) reached 0.514, followed by polymer flooding until the water cut reached 98%. The polymer flooding recovery rate was then calculated, with the results shown in Table 9 and Figure 13. The data indicate that, as the coordination number increases, both the polymer flooding recovery rate and total recovery rate increase.

When the pv reached 0.514, the water cut in the model with a coordination number of 4.3 was 98.78%, with a recovery rate of 31.35%. In the model with a coordination number of 5.4, the water cut was 79.14%, and the recovery rate was 39.99%. For the model with a coordination number of 6, the water cut was 68.72%, and the recovery rate was 44.98%. It is evident that, at the same pv, the recovery rate during water flooding increases as the coordination number increases. The polymer flooding recovery rates for the three models were 24.57%, 27.13%, and 30.44%, respectively. As the coordination number increases, the number of connections between pores and throats also increases, indicating a better connectivity in the model. This enhanced connectivity allows the oil in the pore throats to flow more easily, leading to a higher final recovery rate during polymer flooding.

### 4.2. Study on Residual Oil Distribution and Its Regularity with Different Coordination Numbers

**Figure 14:**
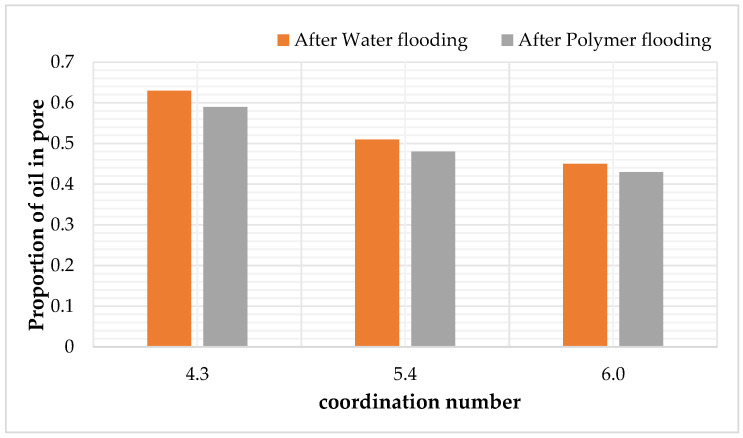
The oil—bearing proportion of pore channels after water—flooding and polymer—flooding under the coordination number scheme.

After water flooding and polymer flooding, the number of pores containing oil was statistically analyzed, and the results are shown in the following Table 10 and Figure 14. Under the pore model conditions with coordination numbers of 4.3, 5.4, and 6, the oil-bearing ratios of the throats after water flooding were 0.58, 0.46, and 0.37, respectively, and the ratios after polymer flooding were 0.56, 0.41, and 0.35, respectively. It can be seen that, as the coordination number increases, the oil-bearing ratio of the throats decreases.

**Figure 15:**
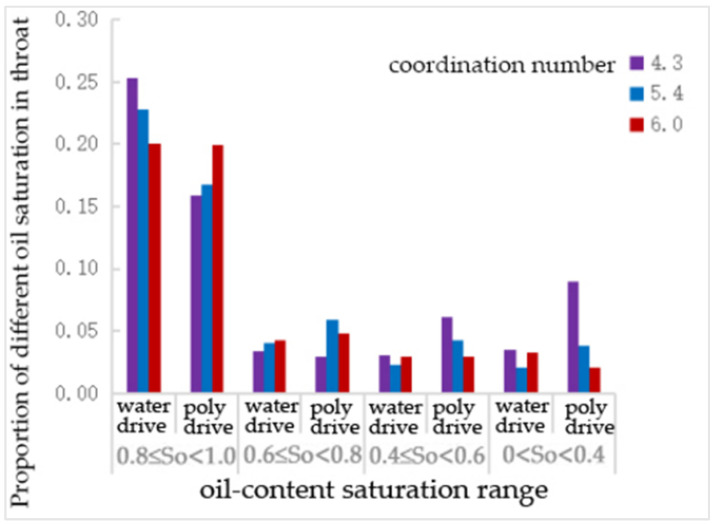
The proportion of different oil—saturation in throats under the coordination—number scheme.

As shown in Table 11 and Figure 15, compared to water flooding, polymer flooding resulted in a 37% and 15% reduction in the number of throats with oil saturation levels of 0.8~1 and 0.6~0.8, respectively, for a coordination number of 4.3. The number of throats with oil saturation levels of 0.4~0.6 and 0~0.4 increased by over 100% and 150%, respectively. For a coordination number of 5.4, the number of throats with an oil saturation of 0.81 decreased by 26%, while the number of throats with oil saturation levels of 0~0.4, 0.4~0.6, and 0.6~0.8 increased by nearly 50%, 80%, and 80%, respectively. For a coordination number of 6.0, the number of throats with an oil saturation of 0~0.4 decreased by 40%, while the number of throats with an oil saturation of 0.6~0.8 increased by 14%.

**Figure 16:**
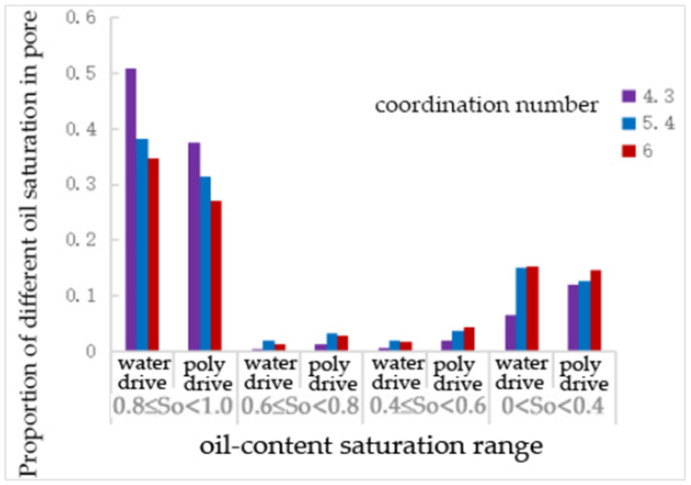
The proportion of pore—different oil saturation under the coordination number scheme.

From Table 12 and Figure 16, it can be seen that, compared to water flooding, polymer flooding resulted in a decrease in the number of pores with an oil saturation of 0.81 in all cases. For a coordination number of 4.3, the number of pores with oil saturation levels of 0.4~0.6 and 0~0.4 increased by 112.5% and 127.9%, respectively. For a coordination number of 5.4, the number of pores with oil saturation levels of 0.6~0.8 and 0.4~0.6 increased by 51.4% and 86.4%, respectively, while the number of pores with an oil saturation of 0~0.4 decreased by 15%. For a coordination number of 6.0, the number of pores with oil saturation levels of 0.6~0.8 and 0.4~0.6 increased by 22.9% and 23.1%, respectively.

**Figure 17:**
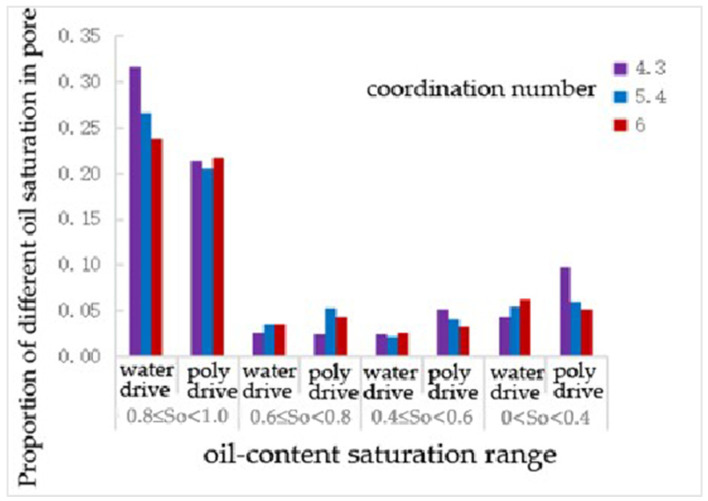
The proportion of different oil saturations of pore channels under the coordination number scheme.

As shown in Table 13 and Figure 17, compared to water flooding, the number of throats with an oil saturation of 0.8~1 decreased after polymer flooding. However, for a coordination number of 6, this decrease was only 9%, which is not as significant compared to the 33% and 23% decreases observed for coordination numbers of 4.3 and 5.4, respectively. The change in the number of throats with an oil saturation of 0.6~0.8 was negligible for a coordination number of 4.3, while it increased by over 50% for a coordination number of 5.4 and then decreased to a 22% increase for a coordination number of 6. The number of throats with an oil saturation of 0.4~0.6 increased by 112%, 86%, and 23% for coordination numbers of 4.3, 5.4, and 6, respectively. The number of throats with an oil saturation of 0–0.4 increased by 128% for a coordination number of 4.3, by 11% for a coordination number of 5.4, and decreased by 17% for a coordination number of 6.0.

### 4.3. Study on Types and Quantitative Analysis of Residual Oil with Different Coordination Numbers

**Figure 18:**
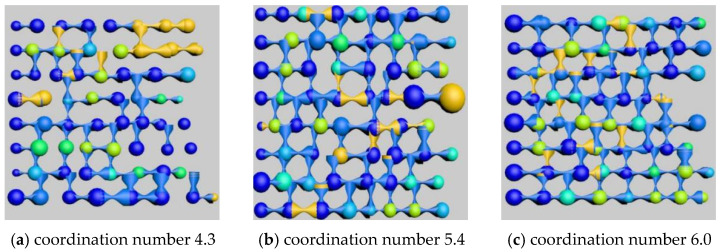
Influence of different coordination number on remaining oil distribution.

**Figure 19:**
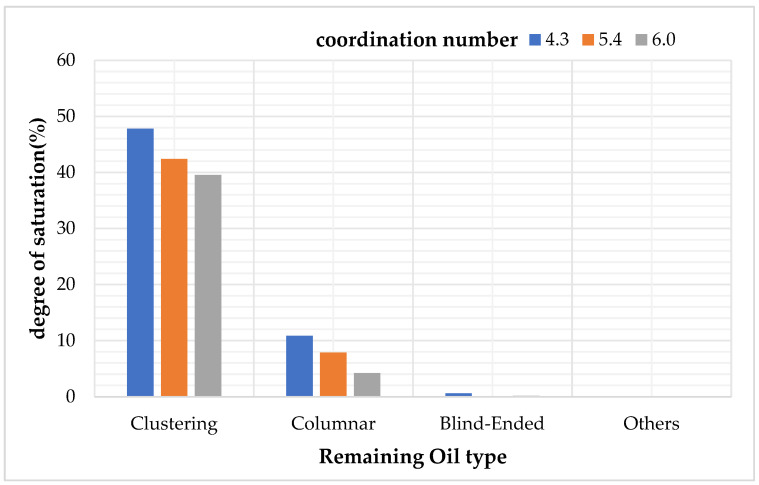
The influence of different coordination number on the type of remaining oil.

After simulating the oil recovery process, the oil–water distribution images of the model cross-sections were exported, as shown in Figure 18. The saturation of each type of residual oil was calculated under the conditions of coordination numbers 4.3, 5.4, and 6, as shown in Table 14 and Figure 19.

The simulation results indicate that, as the coordination number increases, the average number of throats connected to each pore increases, resulting in a higher permeability in the core, making the oil in the pores easier to displace. The data show that the dominant clustered and columnar residual oil types both decrease to varying degrees, while the saturation of other residual oil types, which are present in smaller quantities, shows random variations.

As the coordination number increases, all types of residual oil decrease to varying extents. The more throats there are connected to the pores, the easier it is to displace the residual oil in the pores.

## 5. The Impact of Pore–Throat Ratio on Various Types of Remaining Oil

### 5.1. The Impact of Pore–Throat Ratio on Oil Displacement Efficiency

**Figure 20:**
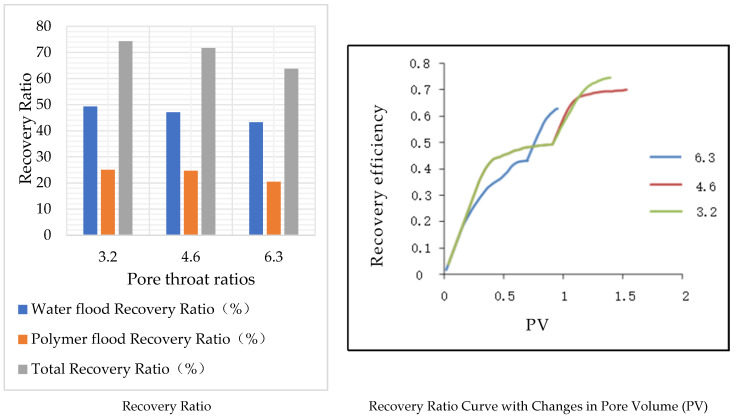
Models of different pore throat ratios.

In this study, three different models with pore–throat ratios of 3.2, 4.6, and 6.3 were selected based on Scheme 2 for displacement simulation. The injection of water and polymer was continued until the water cut at the model outlet consistently reached 0.98. The coordination number, shape factor, and other polymer injection parameters were kept constant. The results are shown in Table 15 and Figure 20.

In the three models, as the pore–throat ratio increases, the simulated permeability of the models decreases to 954.43, 544.12, and 329.05, respectively. When the water saturation reaches 98%, the water flooding recovery, polymer flooding recovery, and total recovery decrease sequentially. This phenomenon can be attributed to the fact that, with the pore radius remaining constant, an increase in the pore–throat ratio results in a reduction in throat radius, leading to an increase in the throat resistance coefficient. Under the same pressure conditions, this makes fluid flow more challenging. Therefore, a higher pore–throat ratio corresponds to a lower recovery ratio.

### 5.2. Study on Residual Oil Distribution and Its Regularity with Different Pore–Throat Ratios

**Figure 21:**
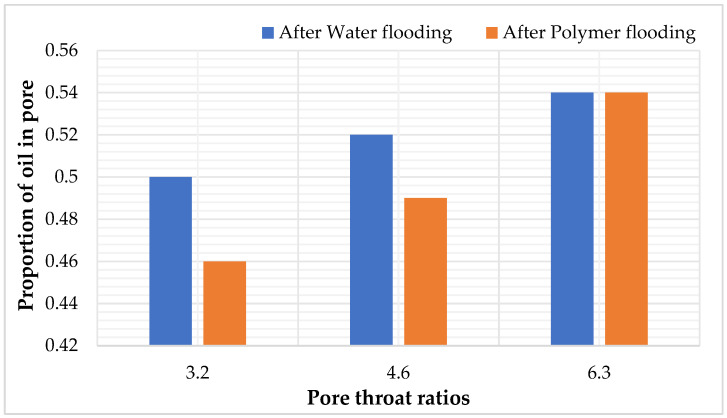
The proportion of oil—bearing in pore channels after water—flooding and polymer—flooding under the pore—throat ratio scheme.

After water flooding and polymer flooding, the number of oil-bearing throats was statistically analyzed using digital pore models. As shown in Table 16 and Figure 21, under the conditions of pore–throat ratios of 3.2, 4.6, and 6.3, the oil-bearing ratios of the throats after water flooding were 0.36, 0.38, and 0.40, respectively, and the ratios after polymer flooding were 0.34, 0.36, and 0.39, respectively. It is evident that, as the pore–throat ratio increases, the oil-bearing ratio of the throats also increases. The pore–throat ratio is inversely proportional to the recovery rate.

**Figure 22:**
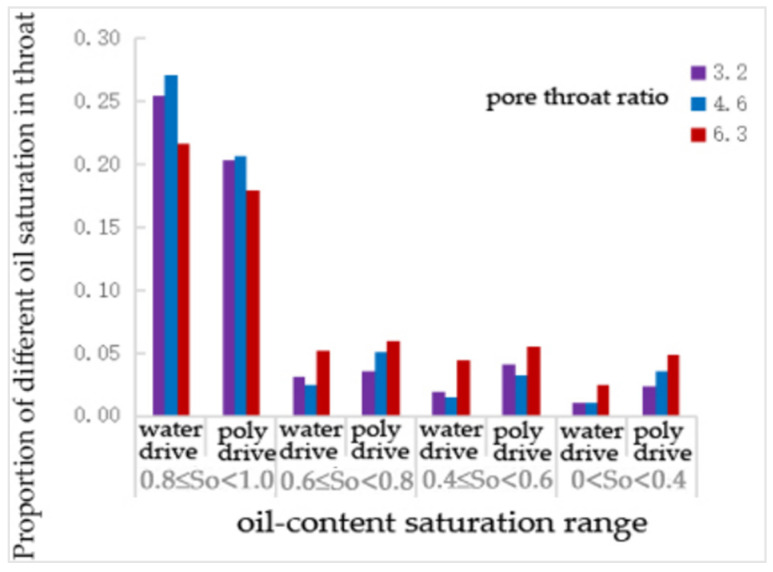
The proportion of different oil—saturation in throats under the pore—throat ratio scheme.

From Table 17 and Figure 22, it can be seen that, compared to water flooding, polymer flooding resulted in varying degrees of reduction in the number of throats with an oil saturation of 0.81. The number of throats with oil saturation levels of 0.6~0.8, 0.4~0.6, and 0~0.4 all increased to different extents. Notably, the number of throats with an oil saturation of 0.6~0.8 increased by 104% at a pore–throat ratio of 4.6. The number of throats with an oil saturation of 0.4~0.6 increased by 116% and 113% at pore–throat ratios of 3.2 and 4.6, respectively. The number of throats with an oil saturation of 0~0.4 increased by 140%, 260%, and 96% at pore–throat ratios of 3.2, 4.6, and 6.3, respectively.

**Figure 23:**
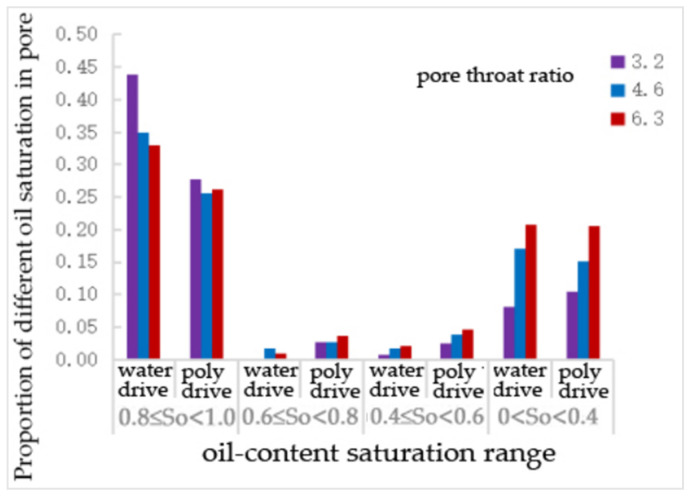
The proportion of pores with different oil saturations under the pore—throat ratio scheme.

Table 18 and Figure 23 shows that, compared to water flooding, polymer flooding resulted in a decrease in the number of pores with an oil saturation of 0.81. The reduction rates at pore–throat ratios of 3.2, 4.6, and 6.3 were 37%, 27%, and 21%, respectively. The number of pores with an oil saturation of 0.6~0.8 increased by 43.5%, 95.7%, and 28.6% at pore–throat ratios of 3.2, 4.6, and 6.3, respectively. The number of pores with an oil saturation of 0.4–0.6 increased by 213%, 117%, and 135% at pore–throat ratios of 3.2, 4.6, and 6.3, respectively. The number of pores with an oil saturation of 0–0.4 increased by 27% at a pore–throat ratio of 3.2, while it decreased by 11% and 1% at pore–throat ratios of 4.6 and 6.3, respectively. The number of pores with zero oil saturation increased by 20%, 18%, and 3% at pore–throat ratios of 3.2, 4.6, and 6.3, respectively.

**Figure 24:**
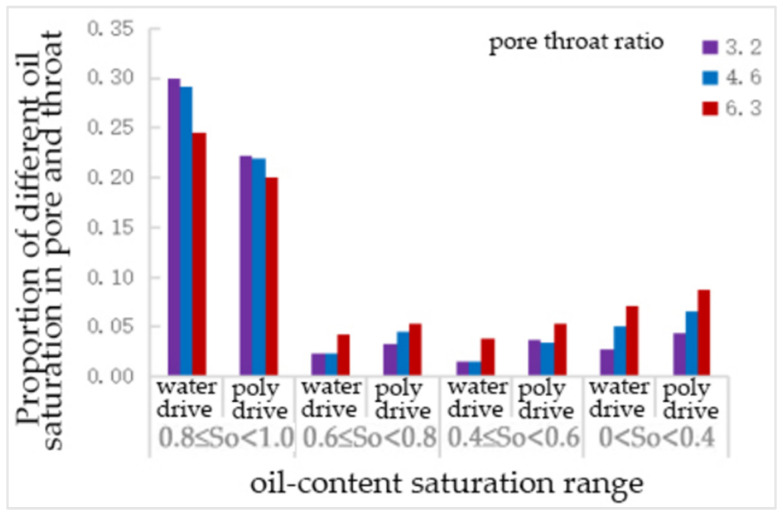
The proportion of pore—throat with different oil saturations under the pore—throat ratio scheme.

From Table 19 and Figure 24, it can be seen that, compared to water flooding, polymer flooding resulted in a certain degree of reduction in the number of throats with an oil saturation of 0.81. The number of throats with an oil saturation of 0.6~0.8 increased by 43%, 96%, and 29% at pore–throat ratios of 3.2, 4.6, and 6.3, respectively. The number of throats with an oil saturation of 0.4–0.6 increased by 131%, 113%, and 29% at pore–throat ratios of 3.2, 4.6, and 6.3, respectively. The number of throats with an oil saturation of 0~0.4 increased by 57%, 30%, and 24% at pore–throat ratios of 3.2, 4.6, and 6.3, respectively. Among these, the increase in the number of throats with an oil saturation of 0.4~0.6 was significantly higher than that of other throats.

### 5.3. Study on Types and Quantitative Analysis of Residual Oil with Different Pore–Throat Ratios

**Figure 25:**
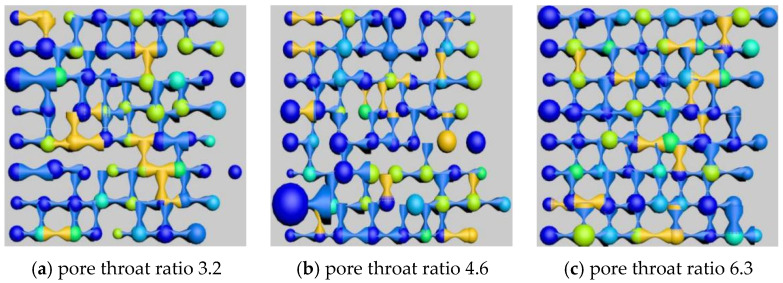
Influence of different pore–throat ratio on remaining oil distribution.

**Figure 26:**
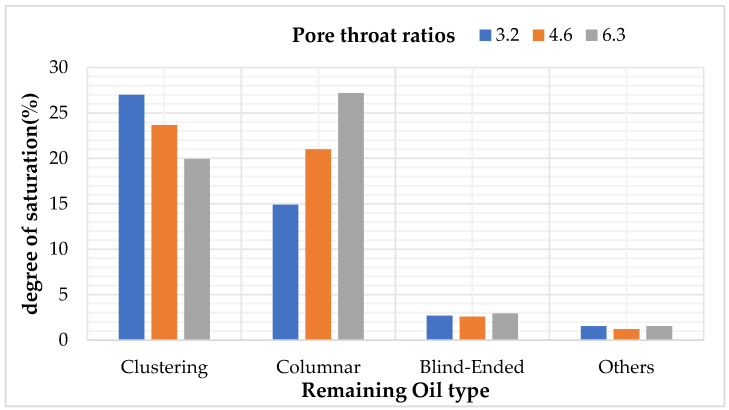
Effects of different pore–throat ratios on remaining oil types.

After simulating polymer flooding in the model, three different models with varying throat ratios were selected to capture and render the distribution of the remaining oil on a specific cross-section, as shown in Figure 25. Subsequently, the remaining oil types and their corresponding saturations in these different throat ratio models were calculated and analyzed, as presented in Table 20 and Figure 26.

The simulation results revealed that the effect of an increasing throat ratio is opposite to the coordination number. When the pore radius remains constant, an increase in the throat ratio results in a reduction in the throat channel radius, naturally leading to a decrease in core permeability. Simultaneously, the throat channel resistance increases, causing more remaining oil to be trapped within the throat channels, resulting in a significant increase in columnar remaining oil.

As the throat ratio decreases, the resistance within the throat channels during displacement decreases, making it less likely for the remaining oil to be trapped inside them. This is manifested by a reduction in columnar remaining oil and an increase in cluster-like remaining oil.

## 6. The Impact of Wettability on Various Types of Remaining Oil

### 6.1. The Impact of Different Wettability Ratios on Oil Displacement Efficiency

**Figure 27:**
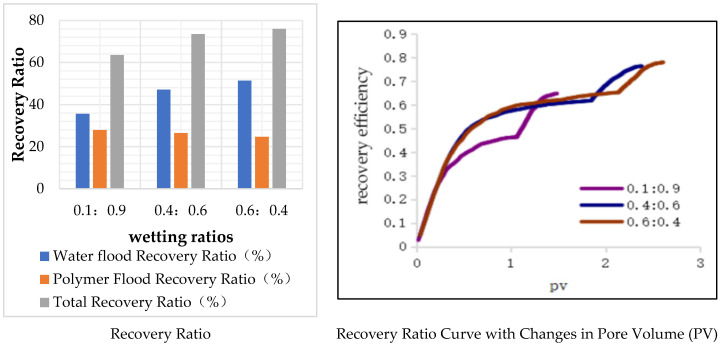
Models with different wetting ratios.

Wettability is a crucial factor in describing the properties of the oil–water interface. In the digital pore model constructed based on Scheme 2, a mixed wettability mode was used, where some throats were water-wet and others were oil-wet. The results of changing the wettability ratio are shown in Table 21 and Figure 27.

In the three models, the ratios of water-wet to oil-wet throats are 0.1:0.9, 0.4:0.6, and 0.6:0.4, respectively. All three models have identical pore structure parameters, resulting in the same porosity. As the proportion of water-wet throats increases, the oil recovery during water flooding increases. Comparing the model with a wetting ratio of 0.1:0.9 to the one with a ratio of 0.6:0.4, the polymer flooding recovery is lower by 0.0326%. However, the corresponding water flooding recovery is higher by 0.0621%, resulting in a total recovery increase of 0.1246%. More water-wet throats are beneficial for oil recovery because, during both water flooding and polymer flooding, if the throats are water-wet, capillary forces act as a driving force, reducing the possibility of the presence of remaining oil. On the other hand, the fewer oil-wet throats there are, the smaller the probability of remaining oil in non-circular throat corners, leading to higher recovery ratios.

### 6.2. Study on Residual Oil Distribution and Its Regularity When Wettability Ratio Is Different

**Figure 28:**
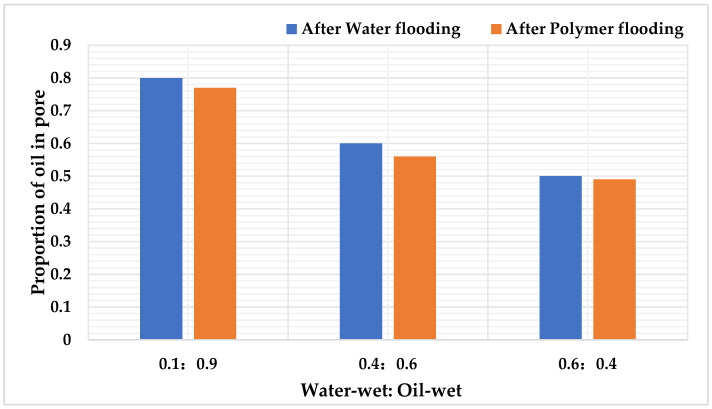
The proportion of oil—bearing in pore channels with different wetting proportions.

After water flooding and polymer flooding in the digital pore model, the numbers of oil-containing pores were separately counted for each scheme. Through Table 22 and Figure 28, it can be analyzed that, under the parameter conditions of wettability ratios at 0.1:0.9, 0.4:0.6, and 0.6:0.4, the oil saturation of pores after water flooding is 0.58, 0.44, and 0.36, respectively, while the oil saturation of pores after polymer flooding is 0.56, 0.41, and 0.35, respectively.

**Figure 29:**
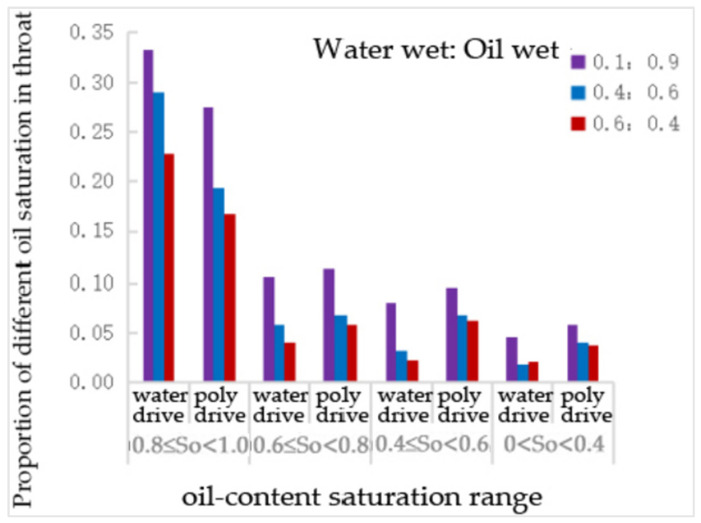
The proportion of different oil—saturation in throats with different wetting proportions.

From Table 23 and Figure 29, it can be observed that, compared to water flooding, polymer flooding resulted in varying degrees of reduction in the number of throats with an oil saturation of 0.81, while the number of throats with other oil saturation levels increased to different extents. Notably, the number of throats with an oil saturation of 0.6~0.8 increased by 8%, 17%, and 48% at wettability ratios of 0.1:0.9, 0.4:0.6, and 0.6:0.4, respectively. The number of throats with an oil saturation of 0.4–0.6 increased by 20%, 106%, and 174%, respectively, under the same conditions. The number of throats with an oil saturation of 0–0.4 increased by 26%, 128%, and 81%, respectively, for these wettability ratios.

**Figure 30:**
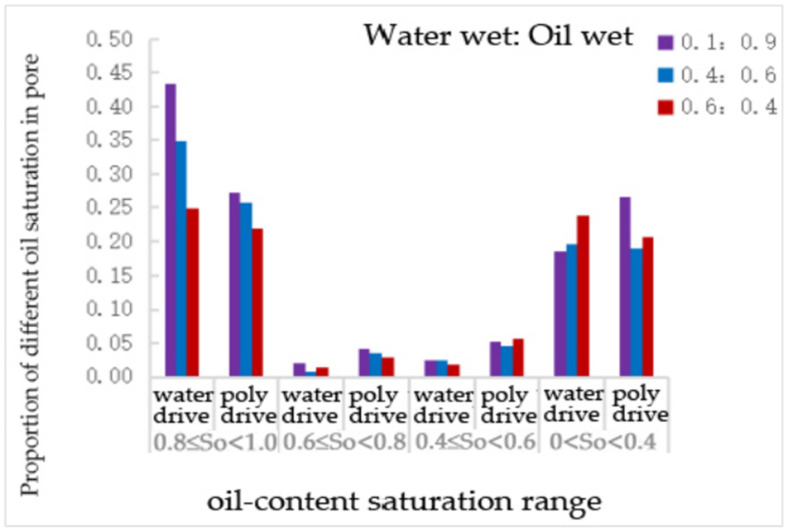
The proportion of different oil saturations in pores with different wetting proportions.

From Table 24 and Figure 30, it can be seen that, compared to water flooding, polymer flooding resulted in varying degrees of reduction in the number of pores with an oil saturation of 0.81, while the number of throats with other oil saturation levels increased, except for a partial reduction in the number of pores with an oil saturation of 0~0.4. The number of pores with an oil saturation of 0.6~0.8 increased by 105%, 338%, and 107% at wettability ratios of 0.1:0.9, 0.4:0.6, and 0.6:0.4, respectively. The number of throats with an oil saturation of 0.4–0.6 increased by 112%, 88%, and 217% under the same conditions. The number of throats with an oil saturation of 0~0.4 increased by 45% at a wettability ratio of 0.1:0.9, while it decreased by 4% and 14% at wettability ratios of 0.4:0.6 and 0.6:0.4, respectively.

**Figure 31:**
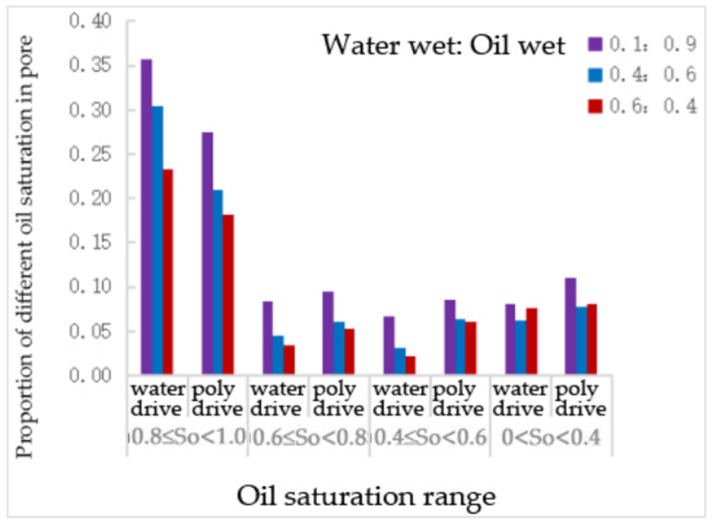
The proportion of different oil—saturation of pore channels with different wetting proportions.

From Table 25 and Figure 31, it can be observed that, compared to water flooding, polymer flooding resulted in varying degrees of reduction in the number of throats with an oil saturation of 0.8~1, while the number of throats with other oil saturation levels increased. The number of throats with an oil saturation of 0.6~0.8 increased by 14%, 33%, and 53% at wettability ratios of 0.1:0.9, 0.4:0.6, and 0.6:0.4, respectively. The number of throats with an oil saturation of 0.4~0.6 increased by 29%, 103%, and 190% under the same conditions. The number of throats with an oil saturation of 0.4 increased by 38%, 26%, and 5% for these wettability ratios.

### 6.3. Study on Types and Quantitative Analysis of Residual Oil When Wettability Ratio Is Different

**Figure 32:**
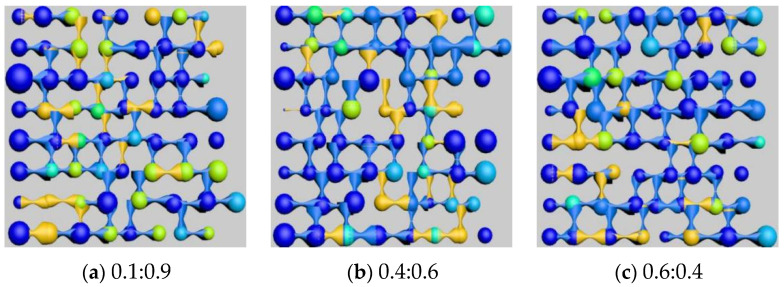
Influence of different wettability on remaining oil distribution.

After simulating polymer flooding, oil distribution images of specific cross-sections were captured and rendered from the models with three different wettability ratios, as shown in Figure 32. The types and corresponding saturation values of residual oil in models with different wettability ratios were then calculated, as shown in Table 26.

**Figure 33:**
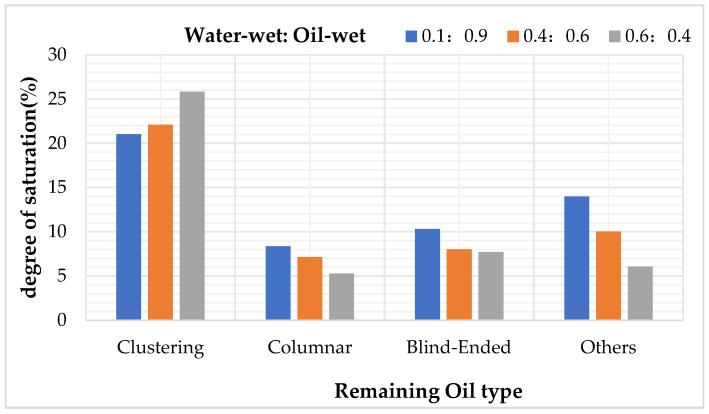
Influence of different wettability on remaining oil type.

The simulation results, as illustrated in Figure 33, indicate that oil-wet models are more likely to form film-like oil due to the adsorption effect of the oil-wet inner walls on the residual oil.

As the wettability shifts from oil-wet to water-wet, the types of residual oil gradually transition from columnar and film-like, which are difficult to displace, to clustered types that are easier to recover. Regardless of whether the conditions are water-wet or oil-wet, the residual oil in the pores predominantly remains in the form of clustered residual oil. However, under neutral wettability conditions, the distribution of residual oil types becomes anomalous, with a higher proportion of droplet-like residual oil. As the wettability shifts from water-wet to oil-wet, the overall trend shows an increase in clustered and film-like residual oil, while the droplet-like and throat-like residual oil decreases. The amount of dead-end residual oil remains stable, indicating that wettability does not affect the presence of dead-end residual oil.

The difference in oil recovery efficiency between composite displacement systems under different throat radii is smaller in water-wet conditions compared to oil-wet conditions. This is because, in water-wet conditions, although the smaller the throat radius, the greater the driving force for oil displacement, the smaller throat radius also increases the likelihood of snap-off, which forms a wetting phase barrier in the advancing oil phase channel, hindering the continued movement of the oil phase. Therefore, the existence of snap-off increases the probability of residual oil formation. However, in oil-wet conditions, the situation is different; the larger the throat radius, the smaller the resistance to oil displacement, and the larger throat radius also reduces the likelihood of snap-off. These two factors together cause the difference in oil recovery efficiency in composite displacement systems to be greater in oil-wet conditions than in water-wet conditions.

## 7. Conclusions

This study investigates the impact of the pore size distribution, coordination number, pore–throat ratio, and wettability on the recovery of microscopic residual oil after polymer flooding, providing theoretical support for the extraction of microscopic residual oil. The research results indicate the following:

(1) Pore Size Distribution: As the distribution of pore size changes, clustered residual oil decreases, while columnar residual oil increases, with other types of residual oil showing random fluctuations. The increase in permeability caused by changes in throat radius makes large clusters of residual oil more likely to be displaced into a more dispersed form.

(2) Coordination Number: As the coordination number increases, the average number of throats connected to each pore increases, leading to a higher permeability of the core and making the oil in the pores easier to displace. The data show that dominant clustered and columnar residual oil types decrease to varying degrees, while the saturation of other less abundant types of residual oil shows random variations due to measurement errors.

(3) Relationship Between Clustered and Columnar Residual Oil: An increase in clustered residual oil is accompanied by a decrease in columnar residual oil. This phenomenon occurs because an increase in circular components at the throat interface can significantly reduce throat resistance, increase core permeability, and make it less likely for residual oil to remain trapped in throat spaces.

(4) Pore–Throat Ratio: The effect of increasing the pore–throat ratio is opposite to that of increasing the coordination number. With a constant pore radius, an increase in the pore–throat ratio results in a decrease in throat radius, which reduces the permeability of the core. At the same time, the throat resistance increases, causing more residual oil to remain in the throats, leading to a significant increase in columnar residual oil.

(5) Wettability Transition: As the wettability shifts from oil-wet to water-wet, the types of residual oil gradually transition from those that are difficult to displace to clustered types that are easier to recover. Regardless of whether the conditions are water-wet or oil-wet, the residual oil in the pores predominantly remains in the form of clustered residual oil. However, under neutral wettability conditions, the distribution of residual oil types becomes anomalous, with a higher proportion of droplet-like residual oil. As the wettability shifts from water-wet to oil-wet, clustered and film-like residual oil types show an overall increase, while droplet-like and throat-like residual oil types decrease. The amount of dead-end residual oil remains stable, indicating that wettability does not affect the presence of dead-end residual oil.

(6) Efficiency of Polymer Flooding Under Different Wettability Conditions: The variation in the oil recovery efficiency of the polymer flooding system under different throat radii is smaller in water-wet conditions compared to oil-wet conditions. This is because, in water-wet conditions, although smaller throat radii result in a greater driving force for oil displacement, they also increase the likelihood of snap-off, creating a wetting phase barrier in the advancing oil phase channel that hinders further oil movement. The presence of snap-off increases the probability of residual oil formation. However, in oil-wet conditions, larger throat radii reduce the resistance to oil displacement, and the likelihood of snap-off also decreases as throat radii increase. The combined effect of these factors leads to a greater variation in oil recovery efficiency in polymer flooding systems under oil-wet conditions compared to water-wet conditions.

## Figures and Tables

**Table 1:** Basic parameters of network model.

Core Number Model Parameter	#1	#2	#3	#4	#5
Porosity (%)	25.1	24.6	24.1	26.3	27.6
Water measurement permeability (10^−3^ μm^2^)	370	39	430	568	623
Length of the larynx (μm)	21~187	3~65	8~155	15~167	16~168
Throat radius (μm)	1~20	1~16	1~18	1~22	1~25
Mean coordination number	4.2	4.3	4.6	4.1	4.2
Average pore–throat ratio	5.4	5.1	4.7	4.3	4.2

**Table 2:** Distribution of throat radius.

Throat Radius (μm)	2	4	6	8	10	12	14	16	18	20	22
#1 distribution frequency	0.002	0.005	0.071	0.279	0.409	0.17	0.047	0.01	0.004	0.002	0.001
#4 distribution frequency	0.001	0.010	0.031	0.092	0.153	0.305	0.194	0.112	0.062	0.030	0.010

**Table 3:** Data validation.

Experiment	Digital Model	Physical Model	Digital Model	Physical Model	Digital Model	Physical Model
Core number porosity (%)	#1 25.6		#3 31.77		#4 27.25	
Permeability (10^−3^ μm^2^)	370		430		568	
Water drive recovery rate (%)	45.03	47.07	46.56	49.12	44.17	46.22
Poly drive recovery rate (%)	23.69	24.65	21.49	22.76	23.68	25.58
Total recovery rate (%)	68.72	71.71	68.05	71.88	67.85	71.76

**Table 4:** Effect of throat radius on polymer flooding recovery.

Scheme	1	2	3
Core ID Porosity (%)	#1 25.6	#3 31.77	#4 27.25
Permeability ($10^{-3}$ μm^2^)	370	430	568
Water flood recovery ratio (%)	47.07	49.12	46.22
Polymer flood recovery ratio (%)	24.65	22.76	25.58
Total recovery ratio (%)	71.71	71.88	71.76

**Table 5:** Oil-bearing ratio of pore channels after water flooding and polymer flooding under the pore size distribution scheme.

Pore Distribution Scheme		Scheme 1	Scheme 2	Scheme 3
Total throat count	Total throat count	993	993	993
	Total pore count	512	512	512
After water flooding	Number of oil-bearing throats	510	480	508
	Number of oil-bearing pores	280	273	284
	Total number of oil-bearing entities	790	753	792
	Oil saturation in pores	0.52	0.50	0.53
After polymer flooding	Number of oil-bearing throats	486	472	496
	Number of oil-bearing pore	275	261	283
	Total number of oil-bearing entities	761	733	779
	Oil saturation in pores	0.51	0.49	0.52

**Table 6:** Proportion of throats with different oil saturations under the pore size distribution scheme.

Oil Saturation	0.8 ≤ So < 1		0.6 ≤ So < 0.8		0.4 ≤ So < 0.6		0 < So < 0.4		0	
Ratio Scheme	Water Flooding	Polymer Flooding	Water Flooding	Polymer Flooding	Water Flooding	Polymer Flooding	Water Flooding	Polymer Flooding	Water Flooding	Polymer Flooding
Scheme 1	0.152	0.110	0.035	0.027	0.021	0.020	0.124	0.159	0.668	0.684
Scheme 2	0.228	0.168	0.040	0.059	0.023	0.042	0.021	0.038	0.688	0.693
Scheme 3	0.165	0.159	0.051	0.029	0.062	0.054	0.039	0.081	0.669	0.677

**Table 7:** The proportion of pores with different oil saturations under the pore—size distribution scheme.

Oil Saturation	0.8 ≤ So < 1		0.6 ≤ So < 0.8		0.4 ≤ So < 0.6		0 < So < 0.4		0	
Ratio Scheme	Water Flooding	Polymer Flooding	Water Flooding	Polymer Flooding	Water Flooding	Polymer Flooding	Water Flooding	Polymer Flooding	Water Flooding	Polymer Flooding
Scheme 1	0.404	0.326	0.006	0.020	0.014	0.018	0.123	0.174	0.453	0.463
Scheme 2	0.348	0.271	0.014	0.029	0.018	0.043	0.154	0.166	0.467	0.490
Scheme 3	0.336	0.303	0.016	0.016	0.025	0.037	0.180	0.197	0.445	0.447

**Table 8:** Influence of different throat radius distribution on remaining oil type.

Type	Saturation (%)		
	Scheme 1	Scheme 2	Scheme 3
Clustering	42.52	37.29	32.05
Columnar	1.99	7.88	14.04
Blind-ended	5.67	0.56	0.01
Others	0.03	1.43	1.49

**Table 9:** Effect of coordination number on recovery efficiency.

Coordination Number	4.3	5.4	6.0
Porosity (%)	25.59	25.86	25.52
Permeability (10^−3^ μm^2^)	243.62	375.36	483.59
Water flood recovery ratio (%)	31.35	39.99	44.98
Polymer flood recovery ratio (%)	24.57	27.13	30.44
Total recovery ratio (%)	55.92	67.12	75.42

**Table 10:** The oil—bearing proportion of pore channels after water—flooding and polymer—flooding under the coordination number scheme.

Coordination Number		4.3	5.4	6.0
Total throat count	Total throat count	822	1016	1120
	Total pore count	512	512	512
After water flooding	Number of oil-bearing throats	541	480	469
	Number of oil-bearing pores	299	292	273
	Total number of oil-bearing entities	840	772	742
	Oil saturation in throats	0.63	0.51	0.45
After polymer flooding	Number of oil-bearing throats	519	472	455
	Number of oil-bearing pores	272	262	251
	Total number of oil-bearing entities	791	734	706
	Oil saturation in throats	0.59	0.48	0.43

**Table 11:** The proportion of different oil—saturation in throats under the coordination—number scheme.

Oil Saturation	0.8 ≤ So < 1		0.6 ≤ So < 0.8		0.4 ≤ So < 0.6		0 < So < 0.4		0	
Ratio Coordination Number	Water Flooding	Polymer Flooding	Water Flooding	Polymer Flooding	Water Flooding	Polymer Flooding	Water Flooding	Polymer Flooding	Water Flooding	Polymer Flooding
4.3	0.253	0.159	0.034	0.029	0.030	0.061	0.035	0.090	0.648	0.662
5.4	0.228	0.168	0.040	0.059	0.023	0.042	0.021	0.038	0.688	0.693
6.0	0.201	0.199	0.042	0.048	0.029	0.029	0.033	0.020	0.695	0.704

**Table 12:** The proportion of pore—different oil saturation under the coordination number scheme.

Oil Saturation	0.8 ≤ So < 1		0.6 ≤ So < 0.8		0.4 ≤ So < 0.6		0 < So < 0.4		0	
Ratio Coordination Number	Water Flooding	Polymer Flooding	Water Flooding	Polymer Flooding	Water Flooding	Polymer Flooding	Water Flooding	Polymer Flooding	Water Flooding	Polymer Flooding
4.3	0.508	0.375	0.004	0.014	0.006	0.021	0.066	0.121	0.416	0.469
5.4	0.381	0.314	0.020	0.033	0.020	0.037	0.150	0.127	0.430	0.488
6.0	0.348	0.271	0.014	0.029	0.018	0.043	0.154	0.146	0.467	0.510

**Table 13:** The proportion of different oil saturations of pore channels under the coordination number scheme.

Ratio Coordination Number	0.8 ≤ So < 1		0.6 ≤ So < 0.8		0.4 ≤ So < 0.6		0 < So < 0.4		0	
Ratio Coordination Number	Water Flooding	Polymer Flooding	Water Flooding	Polymer Flooding	Water Flooding	Polymer Flooding	Water Flooding	Polymer Flooding	Water Flooding	Polymer Flooding
4.3	0.317	0.213	0.026	0.025	0.024	0.051	0.043	0.098	0.590	0.614
5.4	0.266	0.205	0.035	0.053	0.022	0.041	0.054	0.060	0.623	0.642
6.0	0.238	0.217	0.035	0.043	0.026	0.032	0.063	0.052	0.638	0.655

**Table 14:** The influence of different coordination number on the type of remaining oil.

Type	Saturation (%)		
	Coordination Number 4.3	Coordination Number 5.4	Coordination Number 6.0
Clustering	47.79	42.41	39.55
Columnar	10.84	7.89	4.21
Blind-ended	0.61	0.01	0.15
Other	0.01	0.01	0.03

**Table 15:** Effect of pore throat ratio on recovery efficiency.

Pore–Throat Ratio	3.2	4.6	6.3
Porosity (%)	25.47	25.86	25.66
Permeability (10^−3^ μm^2^)	954.43	544.12	329.05
Water flood recovery ratio (%)	49.32	47.07	43.29
Polymer flood recovery ratio (%)	25.02	24.65	20.43
Total recovery ratio (%)	74.34	71.72	63.72

**Table 16:** The proportion of oil—bearing in pore channels after water—flooding and polymer—flooding under the pore—throat ratio scheme.

Pore–Throat Ratio		3.2	4.6	6.3
Total throat count	Total throat count	993	993	993
	Total pore count	512	512	512
After water flooding	Number of oil-bearing throats	482	494	518
	Number of oil-bearing pores	271	284	290
	Total number of oil-bearing entities	753	778	808
	Oil saturation in throats	0.50	0.52	0.54
After polymer flooding	Number of oil-bearing throats	465	501	526
	Number of oil-bearing pores	222	243	282
	Total number of oil-bearing entities	687	742	808
	Oil saturation in throats	0.46	0.49	0.54

**Table 17:** The proportion of different oil—saturation in throats under the pore—throat ratio scheme.

Oil Saturation	0.8 ≤ So < 1		0.6 ≤ So < 0.8		0.4 ≤ So < 0.6		0 < So < 0.4		0	
Ratio of Pore–Throat Ratio	Water Flooding	Polymer Flooding	Water Flooding	Polymer Flooding	Water Flooding	Polymer Flooding	Water Flooding	Polymer Flooding	Water Flooding	Polymer Flooding
3.2	0.255	0.203	0.031	0.035	0.019	0.041	0.010	0.024	0.686	0.697
4.6	0.271	0.207	0.025	0.051	0.015	0.032	0.010	0.036	0.678	0.674
6.3	0.216	0.179	0.052	0.060	0.044	0.055	0.025	0.049	0.663	0.658

**Table 18:** The proportion of pores with different oil saturations under the pore—throat ratio scheme.

Oil Saturation	0.8 ≤ So < 1		0.6 ≤ So < 0.8		0.4 ≤ So < 0.6		0 < So < 0.4		0	
Ratio of Pore–Throat Ratio	Water Flooding	Polymer Flooding	Water Flooding	Polymer Flooding	Water Flooding	Polymer Flooding	Water Flooding	Polymer Flooding	Water Flooding	Polymer Flooding
3.2	0.438	0.277	0.002	0.027	0.008	0.025	0.082	0.104	0.471	0.566
4.6	0.350	0.256	0.018	0.027	0.018	0.039	0.170	0.152	0.445	0.525
6.3	0.330	0.262	0.010	0.037	0.020	0.047	0.207	0.205	0.434	0.449

**Table 19:** The proportion of pore—throat with different oil saturations under the pore—throat ratio scheme.

Oil Saturation	0.8 ≤ So < 1		0.6 ≤ So < 0.8		0.4 ≤ So < 0.6		0 < So < 0.4		0	
Ratio of Pore–Throat Ratio	Water Flooding	Polymer Flooding	Water Flooding	Polymer Flooding	Water Flooding	Polymer Flooding	Water Flooding	Polymer Flooding	Water Flooding	Polymer Flooding
3.2	0.300	0.222	0.023	0.033	0.016	0.037	0.028	0.044	0.632	0.665
4.6	0.291	0.219	0.023	0.045	0.016	0.034	0.050	0.065	0.620	0.637
6.3	0.245	0.200	0.042	0.054	0.038	0.053	0.071	0.088	0.605	0.605

**Table 20:** Effects of different pore–throat ratios on remaining oil types.

Type	Saturation (%)		
	Pore–Throat Ratio 3.2	Pore–Throat Ratio 4.6	Pore–Throat Ratio 6.3
Clustering	27.01	23.67	19.92
Columnar	14.89	20.99	27.19
Blind-ended	2.68	2.57	2.95
Other	1.53	1.21	1.55

**Table 21:** Effect of wettability on recovery efficiency.

Water-Wet:Oil-Wet	0.1:0.9	0.4:0.6	0.6:0.4
Porosity (%)	25.47	25.47	25.47
Water flood recovery ratio (%)	35.63	47.07	51.35
Polymer flood recovery ratio (%)	27.91	26.46	24.65
Total recovery ratio (%)	63.54	73.53	76

**Table 22:** The proportion of oil—bearing in pore channels with different wetting proportions.

Water-Wet:Oil-Wet		0.1:0.9	0.4:0.6	0.6:0.4
Total throat count	Total throat count	993	993	993
	Total pore count	512	512	512
After water flooding	Number of oil-bearing throats	864	611	480
	Number of pil-bearing pores	339	295	265
	Total number of oil-bearing entities	1203	906	745
	Oil saturation in throats	0.80	0.60	0.50
After polymer flooding	Number of oil-bearing throats	832	571	472
	Number of oil-bearing pores	323	269	261
	Total number of oil-bearing entities	1155	840	733
	Oil saturation in throats	0.77	0.56	0.49

**Table 23:** The proportion of different oil—saturation in throats with different wetting proportions.

Oil Saturation	0.8 ≤ So < 1		0.6 ≤ So < 0.8		0.4 ≤ So < 0.6		0 < So < 0.4		0	
Ratio Water-Wet:Oil-Wet	Water Flooding	Polymer Flooding	Water Flooding	Polymer Flooding	Water Flooding	Polymer Flooding	Water Flooding	Polymer Flooding	Water Flooding	Polymer Flooding
0.1:0.9	0.332	0.275	0.105	0.113	0.080	0.096	0.046	0.058	0.438	0.602
0.4:0.6	0.290	0.194	0.058	0.068	0.033	0.068	0.018	0.041	0.602	0.676
0.6:0.4	0.228	0.168	0.040	0.059	0.023	0.063	0.021	0.038	0.688	0.693

**Table 24:** The proportion of different oil saturations in pores with different wetting proportions.

Oil Saturation	0.8 ≤ So < 1		0.6 ≤ So < 0.8		0.4 ≤ So < 0.6		0 < So < 0.4		0	
Ratio Water-Wet:Oil-Wet	Water Flooding	Polymer Flooding	Water Flooding	Polymer Flooding	Water Flooding	Polymer Flooding	Water Flooding	Polymer Flooding	Water Flooding	Polymer Flooding
0.1:0.9	0.434	0.271	0.020	0.041	0.025	0.053	0.184	0.266	0.338	0.369
0.4:0.6	0.348	0.256	0.008	0.035	0.025	0.047	0.195	0.188	0.424	0.475
0.6:0.4	0.248	0.219	0.014	0.029	0.018	0.057	0.238	0.205	0.482	0.490

**Table 25:** The proportion of different oil—saturation of pore channels with different wetting proportions.

Oil Saturation	0.8 ≤ So < 1		0.6 ≤ So < 0.8		0.4 ≤ So < 0.6		0 < So < 0.4		0	
Ratio Water-Wet:Oil-Wet	Water Flooding	Polymer Flooding	Water Flooding	Polymer Flooding	Water Flooding	Polymer Flooding	Water Flooding	Polymer Flooding	Water Flooding	Polymer Flooding
0.1:0.9	0.357	0.274	0.083	0.095	0.066	0.085	0.080	0.110	0.413	0.436
0.4:0.6	0.304	0.209	0.045	0.060	0.031	0.063	0.062	0.078	0.558	0.590
0.6:0.4	0.233	0.181	0.034	0.052	0.021	0.061	0.076	0.080	0.636	0.642

**Table 26:** Influence of different wettability on remaining oil type.

Type Water-Wet:Oil-Wet	Saturation (%)		
	0.1:0.9	0.4:0.6	0.6:0.4
Clustering	21.01	22.11	25.83
Columnar	8.35	7.15	5.27
Blind-ended	10.32	8.01	7.71
Other	13.99	10.03	6.08

## Data Availability

The data are contained within the article.
